# Clinical application of middle descending colon-double lumen ostomy with distal stoma narrowing in the treatment of anorectal malformation

**DOI:** 10.1186/s12887-024-04695-1

**Published:** 2024-03-23

**Authors:** Guoxian Huang, Wenni Li, Lili Ma, Xin Lei, Xiangde Lin, Yuandong Chen, Bo Xu

**Affiliations:** Department of Pediatric Surgery, Women and Children’s Hospital, School of Medicine, Xiamen University, Xiamen, 361000 Fujian China

**Keywords:** Anorectal malformation, Colostomy, Descending colostomy

## Abstract

**Background:**

Anorectal malformations (ARMs) are the most common congenital anomaly of the digestive tract. And colostomy should be performed as the first-stage procedure in neonates diagnosed with intermediate- or high-type ARMs. However, the most classic Pe˜na’s colostomy still has some disadvantages such as complicated operation procedure, susceptibility to infection, a greater possibility of postoperative incision dehiscence, difficulty of nursing and large surgical trauma and incision scarring when closing the stoma. We aimed to explore the effectiveness of middle descending colon-double lumen ostomy (MDCDLO) in the treatment of high and intermediate types of anorectal malformations.

**Methods:**

We retrospectively reviewed the data of patients who underwent MDCDLO for high or intermediate types of ARMs between June 2016 and December 2021 in our hospital. The basic characteristics were recorded. All patients were followed up monthly to determine if any complication happen.

**Results:**

There were 17 boys and 6 girls diagnosed with high or intermediate types of ARMs in our hospital between June 2016 and December 2021. All 23 patients were cured without complications such as abdominal incision infection, stoma stenosis, incisional hernia, and urinary tract infection in the postoperative follow-up time of 6 months to 6 years except one case of proximal intestinal prolapse was restored under anesthesia.

**Conclusion:**

MDCDLO offers the advantages of simplicity, efficiency, safety, mild trauma, and small scarring in the treatment of high and intermediate types of anorectal malformations.

## Background

Anorectal malformations (ARMs) are the most common congenital anomaly of the digestive tract, with an incidence of approximately 3.5 in 10,000 live-births based on a national birth cohort study in England [1]. According to the Krickenbeck diagnosis classification, which combines the advantages of the Wingspread classification with the classification of Pe˜na, ARMs are classified into perineal fistulas, rectourethral fistulas, rectovesical fistulas, vestibular fistulas, cloacal malformations, patients with no fistula, and anal stenosis [2]. Although some scholars suggested that a one-stage operation should be performed in neonates, more scholars advocated that colostomy should be performed first in neonates diagnosed with intermediate- or high- type ARMs,while anorectal reconstructions can be executed 3–6 months later [3, 4]. At present, there are still many controversies over the colostomy site and procedure. Posterior sagittal anorectoplasty (PSARP), known as Pe˜na’s procedure, has been employed to treat patients with ARMs for decades, and it is still preferred by pediatric surgeons. However, the classic Pe˜na’s colostomy, in which there is a transection of the junction of the descending colon and sigmoid colon or transected sigmoid colon and an ostomy with a distal narrowed mucosal fistula, still has the disadvantages of com- plicated operation, susceptibility to infection, a greater possibility of postoperative incision dehiscence, difficulty of nursing and large surgical trauma and incision scarring when closing the stoma [5–7]. We have been performing a one-stage MDCDLO (mid- dle descending colon-double lumen ostomy)that transected the middle segment of the descending colon and performed a double lumen colostomy with the distal colon nar- rowing in the treatment of intermediate and high types of ARMs since 2016. MDCDLO is simple with fewer postoperative complications, easier postoperative nursing care, and satisfactory aesthetic outcomes. This study evaluated the effect of MDCDLO in treating intermediate and high types of ARMs and aimed to provide the clinical basis for its application.

## Materials and methods

### Patients

This retrospective study enrolled 23 patients who underwent MDCDLO for high or intermediate types of ARMs at Women and Children’s Hospital Affiliated with Xiamen University between June 2016 and December 2021. During this time, there were no children who was high or intermediate types of ARMs underwent other approaches. Patients with multiple malformations were excluded. Informed consent was obtained on behalf of the infants by their parents, and this study was approved by the institutional research ethics committee of Women and Children’s Hospital, School of Medicine, Xiamen University.

### Preoperative preparations

All of the patients were prepared preoperatively by fasting, gastrointestinal decompres- sion, rehydration at a dose of 60 80 ml/kg/d, and prophylactic use of a third-generation cephalosporin. Two patients were given respiratory support in the infant radiant warmer. Conventional preoperative tests included routine blood tests, routine urine tests, and biochemical tests; imagings included chest X-rays, invertogram, Doppler echocardiography, urinary Doppler ultrasound, and spinal magnetic resonance imaging.

### Surgical procedures

**Fig. 1:**
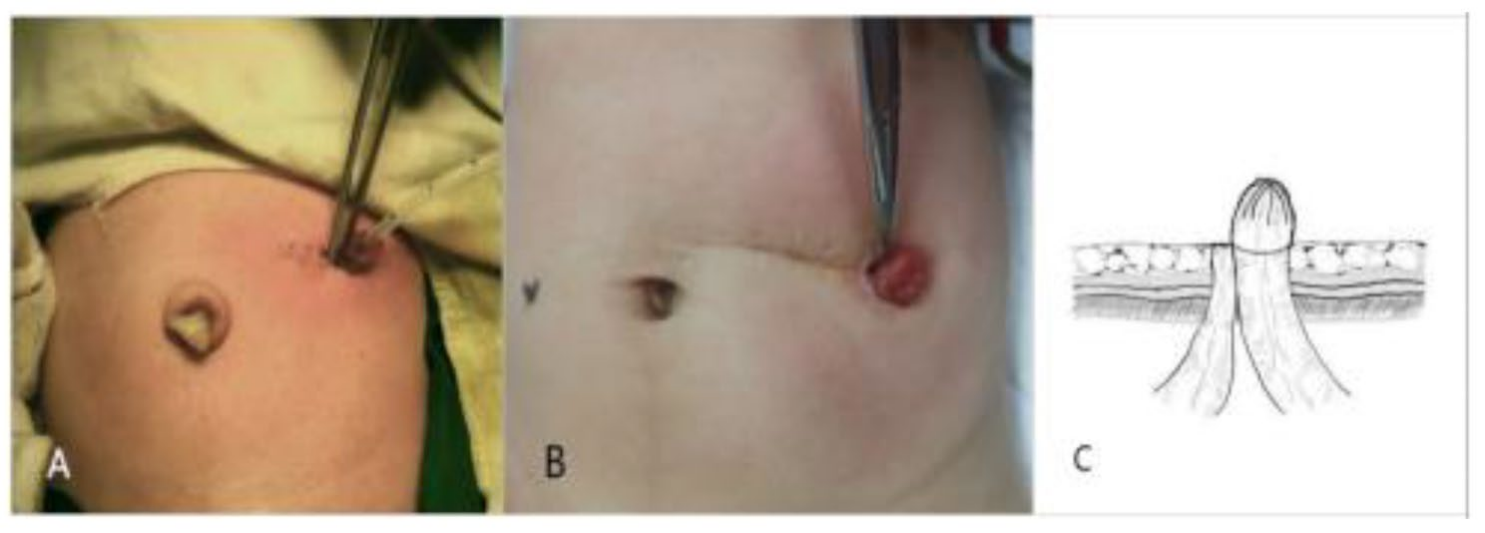
**(A)** A left upper abdominal transverse incision lateral to the rectus abdominis and slightly above the left side of the umbilicus. **(B)** The left upper abdominal stoma and transverse scar three months after MDCDLO. **(C)** The colon was transected in the middle segment of the descending colon after decompression, and the distal stoma was narrowed and sutured under the mucosa of the proximal stoma to form a skin stoma

We performed MDCDLO, in which the middle segment of the descending colon was transected, and performed a double lumen colostomy with the distal colon narrowed to form a stoma (Fig. 1). The surgical procedure was conducted by the same surgeon. We made a transverse incision laterally to the rectus abdominis slightly above the left side of the umbilicus and transected the middle segment of the descending colon after decompression. The distal colon was continuously sutured to reduce the stoma, and then it was sutured layer-by-layer to structure the skin stoma laterally to the incision, thus avoiding distal bowel prolapse. The stoma of the proximal colon was also outside of the incision, adjoined to that of the distal colon. Patients were orally fed 24–48 h after the colostomy.

### Follow-up

Patients were followed up monthly after discharge, and the follow-up team included doctors, nursing staff for fistula maintenance, and family members. Defection and nursing of the stoma as well as complications such as stoma prolapse, stoma stenosis, urinary tract infection, and incisional hernia were observed. Physical assessment and stage II laparoscopic anorectal plasty were performed 26 months after fistulation, and colostomy closure was performed 1-2months after anorectal plasty according to the patient’s situation of anal dilatation. Monthly follow-up was performed until the anus recovered without scarring and independent defecation was good. Then, functional outcomes were evaluated according to Krickenbeck classification. This system encompassed voluntary bowel movements, soiling, and constipation. Voluntary bowel movements was defined as experiencing an urge to defecate, being able to express this sensation verbally, and having the capability to hold the bowel movement. Soiling was classified into three grades as follows: (1) grade 1, occasionally soiling (up to once or twice per week); (2) grade 2, soiling every day but no social problems; and (3) grade 3, constant soiling with social problems, whereas constipation consists of three grades: (1) grade 1, manageable by changes in diet; (2) grade 2, requires laxatives; and (1) grade 3,resistant to laxatives and diet [2].

## Results

**Fig. 2:**
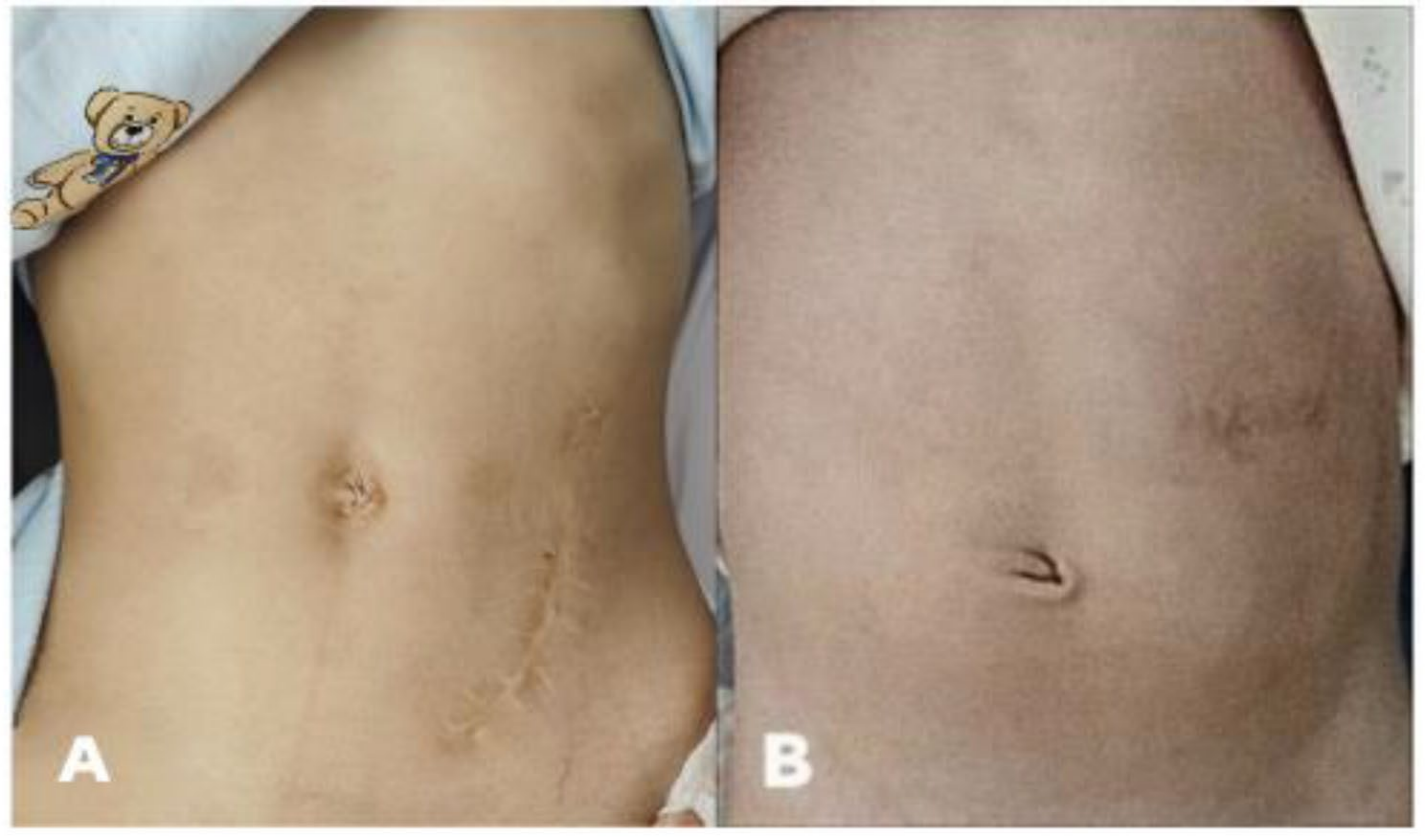
Comparison of postoperative incision scars. **(A)** Oblique incision in anti-mcburney’s point of Peña’s descending colostomy. **(B)** Transverse incision in the left upper abdomen of the MDCDLO

All 23 patients were followed up for 6 months to 6 years, with a mean follow-up period of 3 years and 4 months. There were 17 boys and 6 girls whose mean birth weight was 2930 ± 680 g and mean gestational age was 37.3 ± 2.5 weeks. The patients’ characteristics are shown in Table 1. There were no complications, as abdominal incision infection, stoma stenosis, incisional hernia, or urinary tract infection, but there was 1 case of proximal intestinal prolapse reset under anesthesia. Anoplasty was performed at a mean of 73.7 ± 27.5 days after colostomy, and the stoma was closed at a mean of 43.6 ± 7.7 days after the stage II operation. All of the patients have satisfied functional outcome (Table 2). The MDCDLO led to high satisfaction from the patients’ parents because the left upper abdominal transverse incision along the dermatoglyph led to a smaller incision scar compared with the oblique incision in the anti-mcburney’s point of the sigmoid colon isolated ostomy (Fig. 2).

**Table 1:** Clinical data and follow-up of 23 patients with ARMs

Case	Gender	Gestational Age(week)	Birth weight(g)	Type of ARM†	Follow-up time	Age at MDCDLO (hours)	Stage II surgery (days after MDCLD)	Stoma closure (days after Stage II surgery)
1	M	36 + 3	2960	Rectourethral fistula (Prostatic)	6y	36	61	40
2	F	37 + 5	2950	Cloaca	5y and 10 m	50	99	58
3	M	37 + 1	3060	Rectourethral fistula (Prostatic)	5y and 6 m	38	65	42
4	M	35 + 2	2650	Rectourethral fistula (Bulbar)	5y and 1 m	42	72	45
5	M	37 + 6	3280	Rectourethral fistula (Prostatic)	4y and 9 m	36	56	38
6	M	34 + 6	2280	Rectourethral fistula (Prostatic)	4y and 9 m	46	86	46
7	M	36 + 5	2530	Rectovesical fistula	4y and 5 m	41	80	45
8	M	39 + 6	3580	Rectourethral fistula (Bulbar)	4y and 2 m	37	50	31
9	M	37 + 3	2710	Rectourethral fistula (Prostatic)	3y and 11 m	42	65	42
10	F	38 + 2	3360	No fistula	3y anf 8 m	34	70	45
11	F	37 + 2	2910	Rectovesical fistula	3y and 4 m	45	78	50
12	M	36 + 1	2680	Rectourethral fistula (Bulbar)	3y and 1 m	46	82	52
13	M	35 + 5	2710	Rectourethral fistula (Prostatic)	3y	41	88	46
14	F	36 + 5	2930	No fistula	2y and 11 m	36	66	41
15	M	37 + 4	3105	Rectovesical fistula	2y and 11 m	40	68	30
16	M	36 + 3	2860	Rectovesical fistula	2y and 10 m	44	48	36
17	F	39 + 2	3610	Cloaca	2y and 8 m	56	182	60
18	M	37 + 6	3225	Rectourethral fistula (Prostatic)	2y and 1 m	30	50	32
19	M	35 + 6	2480	Rectourethral fistula (Prostatic)	1y and 11 m	45	81	50
20	F	35 + 3	2390	No fistula	1y and 7 m	32	79	49
21	M	37 + 2	3320	Rectourethral fistula (Bulbar)	1y and 7 m	38	52	39
22	M	38 + 1	3550	Rectourethral fistula (Bulbar)	1y and 1 m	33	48	40
23	M	36 + 6	2730	Rectourethral fistula (Prostatic)	6 m	46	68	46

†This is based on Krickenbeck classification, 2005

**Table 2:** Surgical procedure and functional outcomes

Case	Surgical procedure†	Functional outcomes†		
		Voluntary bowel movements	Soiling	Constipation
1	Laparoscopic-assisted pull-through	Yes	No	No
2	Laparoscopic-assisted pull-through + perineal operation	No	Grade 2	Grade 3
3	Laparoscopic-assisted pull-through	Yes	No	No
4	Laparoscopic-assisted pull-through	Yes	No	No
5	Laparoscopic-assisted pull-through	Yes	No	Grade 1
6	Laparoscopic-assisted pull-through	Yes	No	No
7	Laparoscopic-assisted pull-through	Yes	Grade 3	Grade 2
8	Laparoscopic-assisted pull-through	Yes	No	Grade 2
9	Laparoscopic-assisted pull-through	Yes	Grade 1	No
10	Laparoscopic-assisted pull-through	Yes	Grade 2	Grade 1
11	Laparoscopic-assisted pull-through	Yes	Grade 1	No
12	Laparoscopic-assisted pull-through	Yes	No	No
13	Laparoscopic-assisted pull-through	Yes	No	No
14	Laparoscopic-assisted pull-through	Yes	No	No
15	Laparoscopic-assisted pull-through	Yes	Grade 2	Grade 1
16	Laparoscopic-assisted pull-through	Yes	Grade 1	Grade 2
17	Laparoscopic-assisted pull-through + perineal operation	No	Grade 2	Grade 2
18	Laparoscopic-assisted pull-through	Yes	No	No
19	Laparoscopic-assisted pull-through	Yes	No	Grade 1
20	Laparoscopic-assisted pull-through	Yes	No	No
21	Laparoscopic-assisted pull-through	Yes	Grade 1	Grade 2
22	Laparoscopic-assisted pull-through	Yes	Grade 1	No
23	Laparoscopic-assisted pull-through	NA	NA	NA

†The outcome classification of ARMs is Krickenbeck, 2005

NA: Not applicable

## Discussion

ARMs are the most common congenital anomaly of the digestive tract in newborns. For high and intermediate types of ARMs with urethral fistula, vaginal fistula, blad- der fistula, and cloacal malformation, staging the operations is safer and more feasible. For staging operations, colostomy was performed in the neonatal period, anoplasty was performed at the age of 3–6 months, and stoma closure was executed 1–2 months later [4]. Even though the quality of anoplasty has a lifetime of consequences for patients with ARMs, the colostomy is still critical as the first stage of the staging of operations to solve the problem of defecation in the short period before anoplasty. In addition, by performing a distal colostogram, the distal rectal pouch and abnormal fistulas can be determined, which is beneficial for selecting the appropriate surgical method in the second stage of the operation [8]. Most importantly, the colostomy can effectively reduce the risk of severe infection after anoplasty, thus avoiding anal rec- tal retraction and severe scars that affect the appearance and function of the rebuilt anus. However, in addition to increasing costs and inconvenience, colostomy may also cause serious complications such as stoma prolapse and retraction [9]. Therefore, it is very important to choose the appropriate method of colostomy. However, there have been controversies about the location and mode of colostomy. Improper colostomy will have a serious impact on the whole process of anorectal defect repairment and will also bring unnecessary pain and burden to patients and families [10].

Levitt, Patwardhan, et al. [11, 12] advocated transecting the sigmoid colon and per- forming an ostomy with a distal narrowed mucosal fistula. They believed that the.

operation has the following advantages: 1). It reserved enough of the distal colon for stage II surgery. 2). A separated colostomy can avoid the accumulation of feces in the distal colon,which protects the stoma and ensures distal colon decompression to avoid secondary megarectum. 3). It reduced the chance of urogenital tract infection caused by an abnormal fistula. 4). The distal tube was narrowed to form a skin stoma to avoid stoma retraction and prolapse, and it facilitated distal colostography before the second stage anoplasty to clear the location of the distal rectum and the presence of a fistula. Wu advocated using a separated fistula at the site of the sigmoid colon near the descending colon, the common complications of loop colostomy, such as multiple incisions, wound infection, hydrocolpos (in cloacal malformation), and retrograde uri- nary tract infection, can be avoided. When young parents had difficulty in stoma care or the two ends of the stoma were close to each other during operation, the distal end might not be narrowed to prevent stomal occlusion before stage II anorectoplasty. However, the disadvantages of this type of colostomy were that the oblique incision in the anti-mcburney’s point was long and prone to dehiscence and infection. Further- more, the process of stoma closure is complicated, and the large incision scar causes low aesthetic satisfaction.

Tang advocated loop colostomy in the transverse colon near the splenic flexure [13]. The advantages of this type of colostomy were that the operation was simpler. A small left upper abdominal incision was made to pull out the transverse colon for ostomy, leaving enough distal bowel to form the anus. With the help of the ligament of the splenic flexure, the operated colon can be fixed, reducing the occurrence of stoma pro- lapse. During the loop ostomy, there was no need to divide the mesenteric artery, and the procedure is less invasive. In addition, the stoma closure surgery was less difficult. Furthermore, a stoma in the left upper abdomen was more convenient for nursing, and it did not affect the second-stage laparoscopic surgery. However, too long of the distal colon was lost during transverse colostomy, and the two sections of the intestinal canal could not be completely separated, leading to the accumulation of feces in the distal colon and thus increasing the rate of urinary tract infection in patients with urethral fistula [14]. For children with rectourethral fistula, too long of areserved intes- tine could easily lead to secondary absorption of urine and increase the incidence of hyperchloremic acidosis.

Therefore, we combined the advantages of the loop transverse colostomy and divided sigmoidcolostomy. To avoid the disadvantages of the two types of colostomies as much as possible, we have performed MDCDLO since 2016. At the beginning, in order to make it easier for stoma care, we made incision in the upper left abdomen, and later we found that this location was indeed a little bit high, then we made a transverse incision slightly above the left side of the umbilicus, that is, the proximal stoma location of Pe ~na colostomy.The advantages of MDCDLO were as follows: 1). After transection of the middle part of the descending colon, the reserved distal colon was long enough to ensure the success of the second-stage operation, and the proximal colon was long enough to avoid digestive and absorptive disorders. In patients suffering cloacal malformation, it is necessary to reserve more distal bowel for vaginal reconstruction and anoplasty. Compared with Pe˜na’s colostomy, this method, whose distal mucosal fistula is in the middle or even upper segment of the descending colon, reserves more distal bowel for second-stage surgery [15, 16]. 2). Such an incision does not need to release splenocolic ligament, only the middle of the descending colon needs to be properly released.Colostomy in the middle of the descending colon ensured that the spleen ligament had a fixation effect on the proximal intestinal tube, reducing the occurrence of colon prolapse. 3). The distal stoma was narrowed and sutured under the mucosa of the proximal stoma to form a skin stoma, thus avoiding distal colon fecal accumulation, rectal dilatation, and genitourinary tract infection. 4). We had no difficulty in the distal fistula closure and distal loopogram in practice. The main reasons are as below. During the surgical procedure, the lateral peritoneum is appropriately released to reduce the tension of the distal colon, so that the ostomy can be fixed on the skin without tension. The full thickness of distal loop but not just the mucosa are sutured with the skin to form a small mucosal fistula, which can avoid mucosal retraction and stoma closure.The distal mucocutaneous stoma facilitated contrast examination through the stoma before the second stage anorectoplasty to clear the location of the distal rectum and abnormal fistulas.

5). The operation was more straightforward, and the surgical trauma was minor with a transverse incision of only 2–3 cm long. 6). There was no separation between the proximal and distal stoma, so it was conducive to later stoma closure because extensive dissociation was not needed. 7). The left upper abdomen-located stoma was more convenient for nursing and easier for placing the stoma bags and it did not affect the second-stage laparoscopic surgery. 8) The left upper abdominal transverse incision was along the dermatoglyph, satisfying high aesthetic requirements compared with the oblique incision in the anti-mcburney’s point of divided sigmoid colostomy (Fig. 2). The incision is easy to care, the scar is smaller, and appreciable in appearance.

There were no complications during follow-up for our patients, including abdominal incision infection, stoma retraction, stoma stenosis, incisional hernia, or urinary tract infection In theory, separating the proximal and distal stoma in divided colostomy could prevent the content of the proximal bowel from flowing into the distal bowel and reduce the chance of urogenital tract infection. The above judgment was confirmed by the absence of urogenital tract infection in the follow-up of this study. However, Fouad Youssef believed that the incidence of urinary tract infections of patients with loop colostomy was not significantly higher than that of patients with isolated colostomy, and fear of urinary tract infection alone might not be a compelling reason for divided colostomy [17]. The incidence of stoma prolapse and retraction after divided colostomy and loop colostomy varies among different reports, with that of the divided ostomy reported as 15% 45% and that of the loop ostomy reported as 31% 63% [12, 18, 19]. No complications, such as stoma stenosis, stoma retraction, intestinal mucosal necrosis, and incisional hernia, were observed in this study, and only one case of proximal stoma prolapse occurred without relapse after reduction under anesthesia. All of the patients get satisfied functional outcomes except a one-year-old patient (because of the insufficient follow-up time) according to Krickenbeck functional outcome classification.

Nevertheless, due to the absence of a control group and the retrospective character of the study, this study fails to offer sufficient evidence. In the near future, we will collect information of patients who have undergone alternative methods of colostomy for the purpose of comparing it with the findings presented in this paper.

## Conclusion

In summary, the one-stage operation of middle descending colon-double lumen ostomy has the advantages of simplicity, fewer complications, convenient postoperative nurs- ing, less difficulty for stoma closure surgery, and a small postoperative incision scar in treating high and intermediate ARMs. For staged surgery of ARMs, it can be utilized as a feasible and effective ostomy choice. However, additional study is required to compare the benefits of various approaches.

## Data Availability

The authors confirm that the data supporting the findings of this study are available within the article.

## Abbreviations

ARMs: anorectal malformations
MDCDLO: middle descending colon-double lumen ostomy
PSARP: the posterior sagittal anorectoplasty
